# Supper Timing and Cardiovascular Mortality: The Japan Collaborative Cohort Study

**DOI:** 10.3390/nu13103389

**Published:** 2021-09-27

**Authors:** Jingyun Tang, Jia-Yi Dong, Ehab S. Eshak, Renzhe Cui, Kokoro Shirai, Keyang Liu, Ryoto Sakaniwa, Akiko Tamakoshi, Hiroyasu Iso, on behalf of the JACC Study Group

**Affiliations:** 1 Public Health, Department of Social Medicine, Graduate School of Medicine, Osaka University, 2-2 Yamadaoka, Suita-shi 565-0871, Japan; tjy0603009@gmail.com (J.T.); ehabsalah1@yahoo.com (E.S.E.); cocorosh@gmail.com (K.S.); liukeyang1987@gmail.com (K.L.); ryt.sknw@gmail.com (R.S.); 2 Department of Public Health and Preventive Medicine, Faculty of Medicine, Minia University, Minia 61511, Egypt; 3 Department of Internal Medicine, Okanami General Hospital, 1734 Uenokuwachi, Iga-shi 518-0842, Japan; cuirenzhe@hotmail.com; 4 Department of Public Health, Graduate School of Medicine, Hokkaido University, Kita 15 Jo Nishi 7 Chome, Sapporo-shi 060-8638, Japan; tamaa@med.hokudai.ac.jp

**Keywords:** cohort study, supper time, chrono-nutrition, stroke, stroke type, cardiovascular disease

## Abstract

Evidence on the role of supper timing in the development of cardiovascular disease (CVD) is limited. In this study, we examined the associations between supper timing and risks of mortality from stroke, coronary heart disease (CHD), and total CVD. A total of 28,625 males and 43,213 females, aged 40 to 79 years, free from CVD and cancers at baseline were involved in this study. Participants were divided into three groups: the early supper group (before 8:00 p.m.), the irregular supper group (time irregular), and the late supper group (after 8:00 p.m.). Cox proportional hazards regression models were used to calculate hazard ratios (HRs) for stroke, CHD, and total CVD according to the supper time groups. During the 19-year follow-up, we identified 4706 deaths from total CVD. Compared with the early supper group, the multivariable HR of hemorrhagic stroke mortality for the irregular supper group was 1.44 (95% confidence interval [CI]: 1.05–1.97). There was no significant association between supper timing and the risk of mortality from other types of stroke, CHD, and CVD. We found that adopting an irregular supper timing compared with having dinner before 8:00 p.m. was associated with an increased risk of hemorrhagic stroke mortality.

## 1. Introduction

Cardiovascular disease (CVD) is one of the main causes of premature death worldwide [1] and one of the leading causes of death in Japan from 1990 to 2015 [2]. Irregular eating patterns may have potential adverse effects on cardiometabolic health, obesity, insulin resistance, inflammation, lipid profile, and blood pressure [3,4]. Varied meal timing between weekdays and weekends was reported to be positively associated with BMI in a cross-sectional study that included 1106 students in Spain and Mexico aged 18–25 years [5]. A 16-year follow-up cohort study of 26,902 American males aged 45–82 years found an association between late-night eating and an increased risk of coronary heart disease (CHD) [6]. However, epidemiological data on the association between supper timing and the risk of cardiovascular disease are very limited.

We, therefore, aimed to investigate the association between supper timing and risks of mortality for stroke, CHD, and CVD in middle-aged or older Japanese using a long-term prospective cohort study.

## 2. Materials and Methods

### 2.1. Study Population

**Figure 1:**
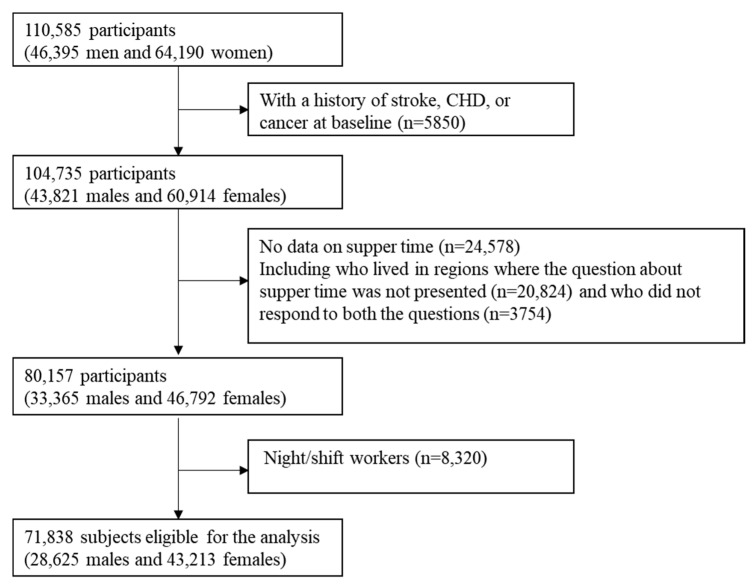
Flowchart of participant selection.

The Japan Collaborative Cohort Study (JACC study), which involved 110,585 subjects (46,395 males and 64,190 females) aged 40–79 years from 45 communities all over Japan, was started in 1988–1990, supported by the Ministry of Education and Science. With informed consent from participants or community leaders, self-administered questionnaires about medical histories and lifestyles were completed by the participants. Details of the JACC study have been described previously [7]. Among all the subjects, 104,735 (43,821 males and 60,914 females) had no history of stroke, CHD, or cancer at baseline. Those who lived in regions where the questionnaire did not include the questions about supper time (*n* = 20,824), those who did not respond to those questions (*n* = 3754), and night/shift workers at baseline (*n* = 8320) were excluded. Accordingly, 71,838 subjects (28,625 males and 43,213 females) were included in this study (Figure 1).

### 2.2. Ethical Approval

The JACC study was approved by the ethics committees of the Graduate Schools of Medicine in Hokkaido University (approval number 14-044) and Osaka University (Approval number 14285).

### 2.3. Dietary Assessment

We used a food-frequency questionnaire (FFQ) [8] containing food and drink intake during the past year without specifying the portion size. There were five possible responses for 33 items: “rarely, 1–2 times/month, 1–2 times/week, 3–4 times/week, and almost every day”, four frequency categories for current alcohol consumers: “<once/week, 1–2 times/week, 3–4 times/week, and ≥5 times/week,” and five frequency categories for tea, green tea, oolong tea, and coffee: “rarely or never, 1–2 cups/month, 1–2 cups/week, 3–4 cups/week, and almost daily (number/day)”. For rice, the categories were: “number of cups/day” and for miso soup: “daily (number/day), every other day, several times a month, and never”. Total energy and nutrient intake were estimated according to the fifth revised edition of the Japan Food Table [9].

The participants were asked the following questions regarding their usual supper timing: 1. “Do you usually eat supper from 5:00 p.m. to 8:00 p.m.? 1. Yes, 2. No”, and those who reported “No” were asked to choose one answer among the following “1. At an irregular time, 2. Always before 5:00 p.m., or 3. Always after 8:00 p.m.”. The participants were divided into three groups according to their responses to the questions about supper timing: the early supper group (those who always eat supper before 8:00 p.m.): including those who chose “yes” and those who chose “no” and “always before 5:00 p.m.”; the irregular supper group (those who eat supper irregularly): those who chose “no” and “at an irregular time”; the late supper group (those who usually eat supper after 8:00 p.m.): those who chose “no” and “always after 8:00 p.m.”. Those who always had supper before 5:00 p.m. and from 5:00 p.m. and 8:00 p.m. were combined as one group, for only 261 subjects (102 males and 159 females) always ate before 5:00 p.m.

### 2.4. Mortality Surveillance

All mortality data were collected by the public health center of the residential area and sent to the Ministry of Health and Welfare, and the causes of death were coded according to the International Classification of Diseases, 10th Revision. Registration of death is required by the Family Registration Law in Japan. Therefore, all deaths that occurred in the cohort were ascertained by death certificates, except for those who died after moving out from their original communities and were treated as censored cases [10]. Cause-specific mortality was coded individually for stroke (I60-I62, I63-I64, I690-I691, I693-I694), hemorrhagic stroke (I60-I62, I690-I692), cerebral infarction (I63, I693), CHD (I20-I25), and total CVD (I01-I99).

### 2.5. Statistical Analysis

Statistical analyses were based on the mortality of each outcome during the follow-up period from 1988–1990 to the end of 2009 (follow-up in 4/45, 4/45, and 2/45 of the areas were ended in 1999, 2003, and 2008). Person-years of follow-up were calculated from the baseline questionnaire (1988–1990) to whichever happened first among the following: time of death, moving out from the original area, and the end of the follow-up.

The age-adjusted *p*-values for the difference in mean values and proportions of characteristics, as well as known risk factors of CVD, were estimated according to supper timing by linear regression for continuous variables and logistic regression for categorical variables. Using the early supper group as the reference category, we estimated age and sex-adjusted HRs and 95% CIs for death from all endpoints through Cox proportional analysis. Then we adjusted further for the history of hypertension and diabetes (yes or no), smoking status (never-smoker, ex-smoker, current smoker consuming 1–19 cigarettes/day, and current smoker consuming ≥20 cigarettes/day), alcohol intake (non-drinker, ex-drinker, current drinker consuming 0.1–11.4 g ethanol/day, 11.5–22.9 g ethanol/day, 23.0–45.9 g ethanol/day, 46.0–68.9 g ethanol/day, and ≥69.0 g ethanol/day), BMI (sex-specific quintiles), hours of walking (rarely or never, <30 min/day, 30–59 min/day, and ≥1 h/day), hours of sports (rarely or never, 1–2 h/week, 3–4 h/week, and ≥5 h/week), hours of sleeping (<6 h/day, 6–7.9 h/day, 8–8.9 h/day, and ≥9 h/day), educational status (<13 years, 13–15 years, 16–18 years, and ≥19 years), perceived mental stress (low, moderate, and high), marital status (married, single, widowed, and divorced) and work schedule (most are day shifts, most are night shifts, and time varied). The third model was further adjusted for food and nutrients: skipping breakfast (yes or no), fresh fish intake (rarely or never, 1–2 times/month, 1–2 times/week, 3–4 times/week, and almost every day), sex-specific quintiles of fruits, vegetables, total energy, sodium, and saturated fatty acids intakes on all endpoints. The selected covariates were the conventional risk factors of CVD and dietary factors that had been shown associated with CVD based on previous reports from the JACC study. The Spearman correlation coefficient between any two variables was <0.70, suggesting that multicollinearity was unlikely. The variables were obtained from the self-administered questionnaires, and some were regrouped or calculated according to the classification criteria. When values of categorical variables were missing, they were treated as a separate category in the models. We tested statistical interactions for skipping breakfast by adding a cross-product term for supper timing (1, 2, and 3) and skipping breakfast (0 and 1) to the model. We conducted stratified analyses by skipping breakfast, BMI ≥ 25 kg/m^2^, and detailed BMI groups (0 < BMI < 18.5 kg/m^2^, 18.5 kg/m^2^ ≤ BMI < 23 kg/m^2^, 23 kg/m^2^ ≤ BMI < 25 kg/m^2^, and BMI ≥ 25 kg/m^2^), and tested the statistical significance for a cross-product term of supper timing and each variable stratifying factor. All *p*-values reported are two-sided, and statistical significance was set at *p* < 0.05. All analyses were performed using SAS version 9.4.

## 3. Results

Among 71,838 participants (28,625 males and 43,213 females) with a median follow-up of 19.2 years, we identified 4706 deaths from total CVD (2337 males and 2369 females); 2044 deaths from stroke (1016 males and 1028 females); 846 deaths from hemorrhagic stroke (379 males and 467 females); 1128 deaths from cerebral infarction (601 males and 527 females), and 954 deaths from CHD (521 males and 433 females). 

The baseline characteristics of the subjects with different supper timing are shown in Table 1. Among all participants, 92.1% (*n* = 66,198) had supper before 8:00 p.m. Compared with the early supper group, people who adopted irregular supper times were more likely to be males, overweight individuals, breakfast skippers, current smokers, and everyday coffee consumers; they were younger, higher educated, and have higher mental stress; they also walked less, had shorter sleep durations, and tended to consume more alcohol, fewer fruits and vegetables, less fish, saturated fatty acids and nearly all food and nutrients. Most of the characteristics of the late supper group were similar to the irregular supper group, while the mean alcohol consumption and the proportions of current smokers and participants walking more than 1 h/day in the late supper group were between those of the other two groups. Besides, the late supper consumers had the highest proportion of well-educated individuals.

The associations between supper timing and death from total stroke, stroke types, CHD, and total CVD are presented in Table 2. In the age- and sex-adjusted model, irregular but not late supper timing was positively associated with risks of hemorrhagic stroke and CVD mortality compared with the early supper group; the HRs (95% CI) were 1.54 (1.13–2.09) and 1.20 (1.02–1.41), respectively for the irregular supper group, and 1.00 (0.58–1.73) and 1.00 (0.77–1.29), respectively for the late supper group. However, after multivariable adjustment, the effects were attenuated. The multivariable HRs were 1.44 (1.05–1.97) for hemorrhagic stroke mortality and 1.12 (0.95–1.32) for total CVD mortality of the irregular supper group, and 0.94 (0.54–1.63) for hemorrhagic stroke mortality and 0.92 (0.71–1.19) for total CVD mortality of the late supper group. When adjusted for age, sex, and breakfast skipping, the HRs of risk from hemorrhagic stroke were 1.52 (95% CI: 1.11–2.08) for the irregular supper group and 0.99 (95% CI: 0.57–1.72) for the late supper group (not shown in the table). No significant association was found between supper timing and the risk of mortality from cerebral infarction, CHD, and total CVD.

We conducted stratified analyses by skipping breakfast and BMI ≥ 25 kg/m^2^, as well as detailed BMI categories, for the proportions of breakfast skippers were significantly higher in irregular supper and late supper consumers than in the early supper consumers, while the prevalence of overweight among irregular supper consumers was the highest. There was no significant interaction by skipping breakfast for all outcome variables (*p* > 0.05). The results among breakfast consumers were similar to those of the main analyses, while the number of breakfast skippers was rather small (Supplementary Table S1).

Stratified analyses by BMI ≥ 25 kg/m^2^ are shown in Supplementary Table S2; there was no interaction by BMI ≥ 25 kg/m^2^ for all outcome variables (*p* > 0.05) other than hemorrhagic stroke (*p* = 0.04). The associations of hemorrhagic stroke mortality among participants with BMI < 25 kg/m^2^ showed similar associations, HRs were 1.55 (95% CI: 1.07–2.23) in the irregular supper group and 1.34 (95% CI: 0.77–2.34) in the late supper group. More detailed BMI stratified analyses are shown in Supplementary Table S3. There was no interaction by the detailed BMI groups for all outcomes (*p* > 0.05). Of note, the association of irregular supper timing became stronger among people with BMI of 23.0 to 24.9 kg/m^2^; the HRs (95% CIs) were 2.15 (1.31–3.53), 2.37 (1.27–4.41), and 1.71 (1.20–2.43) for total stroke, hemorrhagic stroke, and total CVD mortality, respectively.

## 4. Discussion

To the best of our knowledge, our study is the first to investigate the association between supper timing and the risk of cardiovascular mortality. In this large population-based prospective cohort study, after adjusting for CVD risk factors, irregular supper timing was associated with an increased risk of hemorrhagic stroke mortality compared with early supper consumers. We also found positive associations between irregular supper timing and the risk of total stroke, hemorrhagic stroke, and total CVD mortality among subjects with BMI from 23 kg/m^2^ to 24.9 kg/m^2^.

Meal timing is a powerful zeitgeber of peripheral clocks that changes circadian rhythms in metabolism and entrains the central clock of the suprachiasmatic nucleus [11,12]. Later circadian meal timing was associated with higher body fat and BMI [13], and eating meals at irregular times may be associated with oxidative stress [14]. Moreover, the circadian misalignment between the organism and the environment and that among internal tissues are related to cardiometabolic diseases, such as obesity and type 2 diabetes mellitus [11,15,16]. 

In a cross-sectional study of 7081 Korean males aged ≥30 years, participants who had irregular meal timings, compared with regular-time meal consumers, were associated with metabolic syndrome (OR 2.23, 95% CI: 1.60–3.12) [17]. In another cross-sectional study of 1106 students aged 18–25 years in Spain and Mexico, eating jet lag (the eating midpoint on weekends—the eating midpoint on weekdays) was associated with increased BMI (β = 0.283, *p* = 0.008), and having an eating jet lag of more than 3.5 h was associated with 1.34 kg/m^2^ higher BMI (95% CI: 0.026–2.40, *p* = 0.015) [5]. In the same study, dinner jet lag (time on weekends - time on weekdays) was positively associated with chronotype (the midpoint of sleep on free days) (β = 0.073, *p* < 0.00001) and social jet lag (the midpoint of sleep on weekends - the midpoint of sleep on weekdays) (β = 0.072, *p* < 0.001). Chronotype is an important modifier of meal timing associated with obesity [18]. Social jet lag is related to higher consumption of total fat, saturated fat, and cholesterol intake [19], which reflects circadian rhythm disturbances and may increase cardiovascular risk factors, such as obesity and metabolic syndrome [20,21]. In our study, individuals with irregular supper schedules have the highest prevalence of being overweight. Irregular supper timing showed an association with increased risk of hemorrhagic stroke after adjusting for BMI and remained significant in those whose BMI < 25 kg/m^2^. The association was stronger among people with BMI of 23.0 to 24.9 kg/m^2^, which showed associations with a lower risk of total stroke, hemorrhagic stroke, and CVD mortality compared with BMI of less than 18.5 kg/m^2^ in previous reports from the JACC study [22], a similar association was observed between having BMI of 23.0 to 24.9 kg/m^2^ and the risk of CVD mortality among participants with irregular supper time (data not shown). Among subjects with BMI of 23.0 to 24.9 kg/m^2^, irregular supper timing was also associated with increased risks of total stroke and total CVD mortality, implying that irregular supper timing was more likely to be associated with the risk of CVD mortality among people at a lower risk.

In the current study, people who had supper irregularly or after 8:00 p.m. were more likely to be breakfast skippers. In a recent 978-day follow-up cohort using medical records of 1,941,125 working-age Japanese from 2005 to 2018, non-optimal eating behaviors including one, two, or three occasions of skipping breakfast, late-night dinner, and bedtime snacking, compared with the healthy eating behaviors, were positively associated with stroke and heart failure, so were double unhealthy eating behaviors with myocardial infarction, and single or double non-optimal eating behaviors with angina pectoris [23]. A recent meta-analysis of four cohort studies (in Japan and the US) showed that people skipping breakfast were more likely to suffer from an incident of CVD or die from CVD compared with regular breakfast consumers (pooled HR: 1.21, 95% CI: 1.08–1.35; I^2^ = 17.3%, *p* = 0.304) [24]. In these four studies, a 15-year cohort of 82,772 Japanese males and females aged 45–74 years showed that breakfast frequency was inversely associated with the risk of stroke, especially cerebral hemorrhage, the HRs (95% CIs) were 1.14 (1.01–1.27), 1.18 (1.04–1.34), and 1.36 (1.10–1.70) for CVD, stroke, and cerebral hemorrhage, respectively [25]. However, in our sensitivity analysis, the results did not change among breakfast consumers (Supplemental Table S1), while the number of breakfast skippers was too small for analysis.

A 16-year cohort study of 26,902 US males aged 45–82 years showed that late-night eating (eating after going to bed) was associated with an increased risk of CHD (HR: 1.55, 95% CI: 1.05–2.29), although the association became insignificant after further adjustment for potential mediators of BMI and health conditions including diabetes mellitus, hypertension, and hypercholesterolemia (HR: 1.41, 95% CI: 0.95–2.08) [6]. 

Previous studies found that later supper and late-night eating are positively or not associated with risks of overweight/obesity/metabolic syndrome/cardiometabolic profiles and related to unhealthy habits such as smoking and drinking. In a 3.9-year cohort study of 8153 Japanese aged 40–54 years, night eating habits including, “dinner immediately before bed” (dinner within 2 h of bedtime ≥ 3 times/week) and “snacks after dinner” (≥3 times/week) was positively associated with obesity, the HRs were 1.33 (95% CI: 1.02–1.71) and 2.37 (95% CI: 1.71–3.29), respectively [26]. A randomized crossover trial of 20 healthy volunteers aged approximately 26 years, with a fixed sleep duration of 23:00 h-07:00 h showed that late dinner at 22:00 h, compared with routine dinner at 18:00 h, can induce nocturnal glucose intolerance and reduce fatty acid oxidation and mobilization [27]. A large cross-sectional study of 60,800 Japanese aged 20–75 years found that eating dinner within 2 h before bedtime ≥ 3 times/week was associated with increased risk of metabolic syndrome (HR: 1.15, 95% CI: 1.08–1.23), and HRs (95% CIs) of current smoking, daily alcohol consumption, and continuous BMI were 1.16 (1.10–1.22), 2.23 (2.10–2.36), and 1.03 (1.03–1.04), respectively [28]. Another cross-sectional study on Japanese females also showed a positive association of having a late dinner or bedtime snack with the risk of being overweight; the ORs (95% CIs) were 1.43 (1.27–1.62) and 1.47 (1.34–1.62), respectively [29]. However, a 3-year retrospective cohort study of 45,524 Japanese male employees aged 20–49 years showed no association between having dinner within 2 h of bedtime and being overweight; the OR was 0.92 (95% CI: 0.84–1.01) [30]. A study on 432 South-Asian Canadians at risk for diabetes (mean age, 65 years) showed no significant association between dinnertime (<18:00 h, 18:00–20:00 h, or >20:00 h) and cardiometabolic profiles (glycosylated hemoglobin, apolipoprotein, diastolic blood pressure, weight, BMI, and waist circumference) [31]. The varied results in these studies may be due to discrepancies in participant characteristics (including age, sex, race), research design, data collection methods, sample size, and definition of night eating habits.

In the current study, however, late supper was not associated with the risks of stroke, CHD, and CVD, and these results did not change substantially by adjusting for or stratifying by BMI. Eating in the evening or before sleep may be associated with higher BMI through higher calorie intake, but they are not the same, especially for late sleepers [32]. The timing of food intake relative to melatonin onset (circadian evening and/or night) but not the clock hour was associated with the percentage of body fat and BMI [13]. Higher consumption within 2 h before bedtime was associated with being overweight or obese (OR: 1.82; 95% CI: 1.07–3.08), especially in people with a later chronotype (OR: 4.94; 95% CI: 1.61–15.14) in a study of 872 middle-aged or older American by six 24-h dietary recalls in 1 year [18]. Chronotype = the midpoint of time in bed on weekends if the participants slept less on weekends, else chronotype = the midpoint of time in bed on weekends - (the time in bed on weekends - the time in bed on weekdays)/2, and later chronotype was defined using the median cutoff.

Our study design is prospective and population-based, which lowers recall and selection bias. The 19-year median follow-up period allowed us to examine the long-term effect of supper timing on mortality from CVD, stroke, and CHD. In addition, we adjusted the estimated HRs for multiple risk factors associated with the outcomes in our population. Our study had several limitations. First, we had no data on bedtime snacking and the exact bedtime. Second, the lack of repeated measures of dietary habits is a substantial weakness of our study. With the long follow-up period, participants could have changed their dietary habits, including supper timing. However, we assume that supper timing is unlikely to change for individuals without night/shift work and excluded night/shift workers at baseline (*n* = 8320). Third, reverse causation may affect our results. To reduce such bias, we excluded people with histories of CHD, stroke, and cancer at baseline. Sensitivity analysis excluding people dying within the first 5 years attenuated the results; however, the tendency to the increased risk of hemorrhagic stroke with irregular supper timing was still observed [HR: 1.26, 95% CI: 0.86–1.85)] (data not shown). Moreover, although there was a reasonable number of CVD cases in our main analyses, in the stratified analyses, the number of cases limited the validity of the observed associations; however, these secondary analyses should be considered as exploratory analyses. Finally, we considered those having supper after 8:00 p.m. as the late supper time group following previous research on the relation between supper timing and risk of obesity [31,33,34]; however, there is no consensus in defining late supper and late-night eating. To confirm our finding, further studies are needed with detailed information on supper timing as well as other meal timing, along with the exploration of the underlying physiology to explain the role of supper timing in CVD.

## 5. Conclusions

In this prospective cohort study, adopting an irregular supper time, compared with having dinner before 8:00 p.m., was associated with an increased risk of hemorrhagic stroke mortality. We also observed a positive association between irregular supper timing and the risks of stroke, hemorrhagic stroke, and CVD among individuals with a BMI of 23.0 to 24.9 kg/m^2^.

## Figures and Tables

**Table 1:** Baseline characteristics and risk factors (means ± standard deviations) and proportions of participants by supper time.

	Supper Time, p.m.		
	Always ≤8	Irregular	Always >8
No. of subjects	66198	3875	1765
Age (years)	58 ± 10	52 ± 9 ^‡^	52 ± 9 ^‡^
Sex (male %)	39	55 ^‡^	54 ^‡^
Body mass index (kg/m^2^)	22.7 ± 3.0	22.9 ± 3.0	22.8 ± 2.9
Overweight-BMI ≥ 25 kg/m^2^ (%)	20	23 ^‡^	20
BMI < 18.5 kg/m^2^ (%)	6	5	5
Current smokers (%)	24	39 ^‡^	36 ^‡^
Alcohol intake (g/day of ethanol)	28 ± 22	34 ± 26 ^‡^	30 ± 24 *
History of hypertension (%)	22	16	14
History of diabetes mellitus (%)	5	5 ^‡^	4
Sports time ≥ 5 h/week (%)	6	4	4
Walking time ≥ 1 h/day (%)	51	45 ^‡^	48 *
Education level ≥ college (%)	13	17 ^‡^	21 ^‡^
High perceived mental stress (%)	19	39 ^‡^	37 ^‡^
Sleeping time (hours/day)	7.3 ± 1.1	6.9 ± 1.0 ^‡^	6.8 ± 1.0 ^‡^
Energy intake (kcal/day)	1558 ± 456	1547 ± 463 ^†^	1538 ± 455 ^†^
Sodium intake (mg/day)	2017 ± 908	1772 ± 869 ^‡^	1772 ± 854 ^‡^
Potassium intake (mg/day)	2137 ± 614	1971 ± 611 ^‡^	1992 ± 617 ^‡^
Calcium intake (mg/day)	465 ± 156	433 ± 162 ^‡^	444 ± 161 ^‡^
Cholesterol intake (mg/day)	229 ± 89	215 ± 88 ^‡^	218 ± 89 ^‡^
Saturated fatty acids intake(g/day)	9.9 ± 3.7	9.8 ± 3.9 ^‡^	10.2 ± 4.0
N-3 fatty acids intake (g/day)	1.5 ± 0.6	1.3 ± 0.6 ^‡^	1.3 ± 0.6 ^‡^
Vitamin D intake (µg/day)	6.7 ± 3.2	6.1 ± 3.1 ^‡^	6.1 ± 3.1 ^‡^
Fat intake (g/day)	32.4 ± 10.9	30.8 ± 11 ^‡^	31.8 ± 11.2 ^†^
Protein intake (g/day)	54 ± 15	50 ± 15 ^‡^	50 ± 15 ^‡^
Carbohydrate intake (g/day)	238 ± 75	225 ± 75 ^‡^	225 ± 76 ^‡^
Total dietary fiber intake (g/day)	12.4 ± 3.8	11.1 ± 3.7 ^‡^	11.1 ± 3.7 ^‡^
Vegetable intake (g/day)	276 ± 322	226 ± 298 ^‡^	269 ± 321
Fruit intake (g/day)	88 ± 52	79 ± 53 ^‡^	78 ± 53 ^‡^
Miso soup every day (%)	71	60 ^‡^	61 ^‡^
Other soy products intake (g/day)	40 ± 24	33 ± 22 ^‡^	35 ± 23 ^‡^
Seaweed intake (g/day)	3.8 ± 1.0	3.6 ± 1.0 ^‡^	3.6 ± 1.1 ^‡^
Milk and dairy products intake (g/day)	92 ± 72	85 ± 71 ^‡^	87 ± 72
Meat intake (g/day)	29 ± 20	29 ± 21 ^‡^	29 ± 21
Total seafood intake (g/day)	50 ± 28	45 ± 27 ^‡^	45 ± 27 ^‡^
Fresh fish intake (g/day)	31 ± 21	29 ± 21 ^‡^	28 ± 20 ^‡^
Egg intake (mean times/week)	4.3 ± 2.5	4.1 ± 2.5 ^‡^	4.2 ± 2.6 ^†^
Coffee intake every day (%)	50	68 ^‡^	69 ^‡^
Green tea intake every day (%)	84	80 ^‡^	81 *
Marital status (married %)	88	88 ^‡^	90 ^†^
Skipping breakfast (%)	2	8 ^‡^	8 ^‡^

* *p* < 0.05; ^†^ *p* < 0.01; ^‡^ *p* < 0.001. *p* for all the variables other than age are age-adjusted. The age-adjusted *p*-values were estimated by the regression method (linear regression for continuous variables and logistic regression for categorical variables).

**Table 2:** Hazard ratios (HRs) and 95% confidence intervals (CIs) for cardiovascular mortality outcomes according to supper time.

	Supper Time, p.m.		
	Always ≤8	Irregular	Always >8
No. of subjects	66,198	3875	1765
Person-years	1,072,692	63,451	29,514
Total stroke, *n*	1952	69	23
Age and sex-adjusted HR (95% CI)	1	1.23 (0.97–1.57)	0.87 (0.58–1.32)
Multivariable HR (95% CI) *	1	1.16 (0.91–1.48)	0.82 (0.54–1.24)
Multivariable HR (95% CI) ^†^	1	1.16 (0.91–1.48)	0.81 (0.53–1.22)
Cerebral infarction, *n*	1095	23	10
Age and sex-adjusted HR (95% CI)	1	0.86 (0.57–1.30)	0.78 (0.42–1.46)
Multivariable HR (95% CI) *	1	0.80 (0.53–1.21)	0.73 (0.39–1.37)
Multivariable HR (95% CI) ^†^	1	0.81 (0.53–1.23)	0.71 (0.38–1.32)
Hemorrhagic stroke, *n*	790	43	13
Age and sex--adjusted HR (95% CI)	1	1.54 (1.13–2.09)	1.00 (0.58–1.73)
Multivariable HR (95% CI) *	1	1.43 (1.05–1.96)	0.94 (0.54–1.63)
Multivariable HR (95% CI) ^†^	1	1.44 (1.05–1.97)	0.94 (0.54–1.63)
Coronary heart disease, *n*	908	32	14
Age and sex-adjusted HR (95% CI)	1	1.19 (0.83–1.69)	1.10 (0.65–1.87)
Multivariable HR (95% CI) *	1	1.05 (0.74–1.50)	1.02 (0.60–1.73)
Multivariable HR (95% CI) ^†^	1	1.04 (0.72–1.48)	0.99 (0.58–1.69)
Total cardiovascular disease, *n*	4492	154	60
Age and sex-adjusted HR (95% CI)	1	1.20 (1.02–1.41)	1.00 (0.77–1.29)
Multivariable HR (95% CI) *	1	1.13 (0.96–1.33)	0.94 (0.73–1.22)
Multivariable HR (95% CI) ^†^	1	1.12 (0.95–1.32)	0.92 (0.71–1.19)

* Adjusted further for histories of hypertension and diabetes, smoking status, alcohol intake, BMI, hours of walking, hours of sports, hours of sleeping, educational status, perceived mental stress, and marital status. ^†^ Adjusted further for food and nutrients: skipping breakfast, total energy intake, food intakes of fish, fruits, vegetables, and nutrients intakes of sodium and saturated fatty acids.

## Data Availability

Data was obtained from the JACC study group and are available at https://publichealth.med.hokudai.ac.jp/jacc/ (accessed on 11 August 2021) with the permission of the JACC study group.

## Supplementary Materials

The following are available online at https://www.mdpi.com/article/10.3390/nu13103389/s1, Table S1: Hazard ratios (HRs) and 95% confidence intervals (CIs) for cardiovascular mortality outcomes according to supper time among breakfast skippers and breakfast consumers, Table S2: BMI stratified hazard ratios (HRs) and 95% confidence intervals (CIs) for cardiovascular mortality outcomes according to supper time, Table S3: BMI stratified hazard ratios (HRs) and 95% confidence intervals (CIs) for cardiovascular mortality outcomes according to supper time.

## Abbreviations

BMI, body mass index; CI, confidence interval; CHD, coronary heart disease; CVD, cardiovascular disease; FFQ, food frequency questionnaire; HR, hazard ratio; the JACC study, The Japan Collaborative Cohort Study; OR, odds ratio.
