# Supplementary material for: Supper Timing and Cardiovascular Mortality: The Japan Collaborative Cohort Study
Source: Nutrients. 2021 Sep 27;13(10):3389. doi: 10.3390/nu13103389 (PMC8541292; doi:10.3390/nu13103389)
Supplement: Supplementary file 1 [file nutrients-13-03389-s001.zip › nutrients-1358225-supplementary.pdf]

nutrients

MDPI

Supplementary Materials

Supplementary Table S1 Hazard ratios (HRs) and 95% confidence intervals (CIs) for cardiovascular mortality outcomes according to supper time among breakfast skippers and breakfast consumers.

	Supper time, pm		
	Always ≤8	Irregular	Always >8
**Breakfast skippers, n**	1167	302	132
Person-years	18963	4958	2216
**Total stroke, n**	29	4	2
Age and sex-adjusted HR (95% CI)	1	———	———
Multivariable HR (95% CI)*	1	———	———
Multivariable HR (95% CI)†	1	———	———
**Cerebral infarction, n**	11	1	1
Age and sex-adjusted HR (95% CI)	1	———	———
Multivariable HR (95% CI)*	1	———	———
Multivariable HR (95% CI)†	1	———	———
**Hemorrhagic stroke, n**	10	3	0
Age and sex-adjusted HR (95% CI)	1	———	———
Multivariable HR (95% CI)*	1	———	———
Multivariable HR (95% CI)†	1	———	———
**Coronary heart disease, n**	16	3	1
Age and sex-adjusted HR (95% CI)	1	———	———
Multivariable HR (95% CI)*	1	———	———
Multivariable HR (95% CI)†	1	———	———
**Total cardiovascular disease, n**	60	12	4
Age and sex-adjusted HR (95% CI)	1	1.26(0.67-2.36)	0.74(0.27-2.05)
Multivariable HR (95% CI)*	1	1.39(0.69-2.82)	1.16(0.40-3.35)
Multivariable HR (95% CI)†	1	1.14(0.51-2.51)	1.11(0.36-3.39)
**Breakfast consumers, n**	64457	3511	1608
Person-years	1044943	57491	26898
**Total stroke, n**	1900	65	21
Age and sex-adjusted HR (95% CI)	1	1.27(0.99-1.62)	0.88(0.57-1.35)
Multivariable HR (95% CI)*	1	1.19(0.92-1.52)	0.82(0.53-1.26)
Multivariable HR (95% CI)†	1	1.20(0.93-1.54)	0.81(0.53-1.25)
**Cerebral infarction, n**	1068	22	8
Age and sex-adjusted HR (95% CI)	1	0.89(0.59-1.37)	0.69(0.35-1.38)
Multivariable HR (95% CI)*	1	0.82(0.54-1.26)	0.63(0.32-1.27)
Multivariable HR (95% CI)†	1	0.84(0.55-1.28)	0.62(0.31-1.25)
**Hemorrhagic stroke, n**	768	40	13
Age and sex-adjusted HR (95% CI)	1	1.56(1.13-2.15)	1.09(0.63-1.90)
Multivariable HR (95% CI)*	1	1.47(1.06-2.03)	1.03(0.60-1.79)
Multivariable HR (95% CI)†	1	1.47(1.06-2.04)	1.03(0.59-1.79)
**Coronary heart disease, n**	883	27	13
Age and sex-adjusted HR (95% CI)	1	1.10(0.75-1.61)	1.13(0.65-1.95)
Multivariable HR (95% CI)*	1	0.98(0.66-1.44)	1.03(0.60-1.79)
Multivariable HR (95% CI)†	1	0.97(0.66-1.44)	1.03(0.59-1.78)
**Total cardiovascular disease, n**	4376	137	55

Age and sex-adjusted HR (95% CI)	1	1.17(0.98-1.38)	1.01(0.77-1.31)
Multivariable HR (95% CI)*	1	1.09(0.92-1.30)	0.94(0.72-1.23)
Multivariable HR (95% CI)†	1	1.10(0.92-1.30)	0.93(0.71-1.22)

* Adjusted further for histories of hypertension and diabetes, smoking status, alcohol intake, BMI, hours of walking, hours of sports, hours of sleeping, educational status, perceived mental stress, and marital status.

† Adjusted further for food and nutrients: total energy intake, food intakes of fish, fruits, vegetables, and nutrients intakes of sodium and saturated fatty acids.

Supplementary Table S2 BMI stratified hazard ratios (HRs) and 95% confidence intervals (CIs) for cardiovascular mortality outcomes according to supper time.

	Supper time, pm		
	Always ≤8	Irregular	Always >8
**BMI ≥25 kg/m^2, n**	12228	840	336
Person-years	203325	13836	5773
**Total stroke, n**	324	9	2
Age and sex-adjusted HR (95% CI)	1	0.78(0.40-1.52)	0.38(0.10-1.54)
Multivariable HR (95% CI)*	1	0.77(0.39-1.52)	0.39(0.10-1.57)
Multivariable HR (95% CI)†	1	0.77(0.39-1.51)	0.37(0.09-1.48)
**Cerebral infarction, n**	178	4	2
Age and sex-adjusted HR (95% CI)	1	————	————
Multivariable HR (95% CI)*	1	————	————
Multivariable HR (95% CI)†	1	————	————
**Hemorrhagic stroke, n**	134	5	0
Age and sex-adjusted HR (95% CI)	1	0.84(0.34-2.06)	————
Multivariable HR (95% CI)*	1	0.85(0.34-2.12)	————
Multivariable HR (95% CI)†	1	0.87(0.35-2.17)	————
**Coronary heart disease, n**	190	7	4
Age and sex-adjusted HR (95% CI)	1	0.88(0.41-1.89)	1.14(0.42-3.08)
Multivariable HR (95% CI)*	1	0.84(0.39-1.83)	1.28(0.47-3.51)
Multivariable HR (95% CI)†	1	0.86(0.39-1.86)	1.21(0.44-3.34)
**Total cardiovascular disease, n**	780	26	12
Age and sex-adjusted HR (95% CI)	1	0.92(0.62-1.37)	0.94(0.53-1.66)
Multivariable HR (95% CI)*	1	0.92(0.61-1.36)	0.96(0.54-1.72)
Multivariable HR (95% CI)†	1	0.90(0.60-1.34)	0.93(0.52-1.66)
**BMI <25 kg/m^2, n**	50247	2853	1334
Person-years	815097	46911	22284
**Total stroke, n**	1392	48	19
Age and sex-adjusted HR (95% CI)	1	1.26(0.94-1.68)	1.03(0.65-1.62)
Multivariable HR (95% CI)*	1	1.20(0.90-1.61)	0.98(0.62-1.55)
Multivariable HR (95% CI)†	1	1.21(0.90-1.62)	0.96(0.61-1.52)
**Cerebral infarction, n**	778	14	6
Age and sex-adjusted HR (95% CI)	1	0.79(0.47-1.35)	0.67(0.30-1.50)
Multivariable HR (95% CI)*	1	0.76(0.44-1.29)	0.63(0.28-1.42)
Multivariable HR (95% CI)†	1	0.77(0.45-1.31)	0.60(0.27-1.35)
**Hemorrhagic stroke, n**	565	32	13
Age and sex-adjusted HR (95% CI)	1	1.65(1.15-2.37)	1.41(0.81-2.46)
Multivariable HR (95% CI)*	1	1.54(1.07-2.22)	1.35(0.78-2.36)

Multivariable HR (95% CI)†	1	1.55(1.07-2.23)	1.34(0.77-2.34)
**Coronary heart disease, n**	626	24	9
Age and sex-adjusted HR (95% CI)	1	1.42(0.94-2.13)	1.09(0.56-2.10)
Multivariable HR (95% CI)*	1	1.25(0.83-1.90)	1.00(0.52-1.94)
Multivariable HR (95% CI)†	1	1.21(0.80-1.84)	0.98(0.51-1.91)
**Total cardiovascular disease, n**	3195	109	43
Age and sex-adjusted HR (95% CI)	1	1.28(1.05-1.55)	1.04(0.77-1.40)
Multivariable HR (95% CI)*	1	1.20(0.99-1.46)	0.98(0.73-1.33)
Multivariable HR (95% CI)†	1	1.20(0.99-1.45)	0.96(0.71-1.30)

* Adjusted further for histories of hypertension and diabetes, smoking status, alcohol intake, hours of walking, hours of sports, hours of sleeping, educational status, perceived mental stress, and marital status.

† Adjusted further for food and nutrients: skipping breakfast, total energy intake, food intakes of fish, fruits, vegetables, and nutrients intakes of sodium and saturated fatty acids.

Supplementary Table S3 BMI stratified hazard ratios (HRs) and 95% confidence intervals (CIs) for cardiovascular mortality outcomes according to supper time.

	**0<BMI<18.5 kg/m²**			**18.5 kg/m²≤BMI< 23kg/m²**			**23 kg/m²≤BMI<25 kg/m²**			**BMI ≥25 kg/m²**		
**Supper time, pm**	Always ≤8	Irregular	Always >8	Always ≤8	Irregular	Always >8	Always ≤8	Irregular	Always >8	Always ≤8	Irregular	Always >8
**Number of subjects, n**	3893	185	80	31711	1794	844	14643	874	410	12228	840	336
Person-years	55683	2761	1194	515919	29710	14011	243495	14441	7080	203325	13836	5773
**Total stroke, n**	206	6	4	855	24	12	331	18	3	324	9	2
Age and sex-adjusted HR (95% CI)	1	1.11(0.49-2.51)	1.73(0.64-4.66)	1	0.99(0.66-1.49)	1.07(0.61-1.90)	1	2.07(1.28-3.35)	0.60(0.19-1.88)	1	0.78(0.40-1.52)	0.38(0.10-1.54)
Multivariable HR (95% CI)*	1	1.00(0.44-2.29)	1.73(0.63-4.71)	1	0.95(0.63-1.43)	1.01(0.57-1.79)	1	2.10(1.29-3.43)	0.61(0.19-1.91)	1	0.77(0.39-1.52)	0.39(0.10-1.57)
Multivariable HR (95% CI)†	1	1.07(0.46-2.48)	1.70(0.61-4.73)	1	0.95(0.63-1.44)	0.96(0.54-1.70)	1	2.15(1.31-3.53)	0.68(0.22-2.13)	1	0.77(0.39-1.51)	0.37(0.09-1.48)
**Cerebral infarction, n**	107	2	1	491	6	4	180	6	1	178	4	2
Age and sex-adjusted HR (95% CI)	1	————	————	1	0.50(0.23-1.13)	0.71(0.27-1.91)	1	1.77(0.78-4.01)	————	1	————	————
Multivariable HR (95% CI)*	1	————	————	1	0.48(0.21-1.08)	0.66(0.25-1.78)	1	1.92(0.84-4.41)	————	1	————	————
Multivariable HR (95% CI)†	1	————	————	1	0.48(0.21-1.09)	0.58(0.21-1.56)	1	2.01(0.87-4.67)	————	1	————	————
**Hemorrhagic stroke, n**	88	3	3	337	17	8	140	12	2	134	5	0
Age and sex-adjusted HR (95% CI)	1	————	————	1	1.43(0.87-2.34)	1.46(0.72-2.96)	1	2.38(1.30-4.34)	0.76(0.19-3.10)	1	0.84(0.34-2.06)	————
Multivariable HR (95% CI)*	1	————	————	1	1.34(0.81-2.20)	1.36(0.67-2.77)	1	2.31(1.25-4.27)	0.81(0.20-3.30)	1	0.85(0.34-2.12)	————
Multivariable HR (95% CI)†	1	————	————	1	1.35(0.81-2.22)	1.36(0.67-2.77)	1	2.37(1.27-4.41)	0.85(0.21-3.48)	1	0.87(0.35-2.17)	————
**Coronary heart disease, n**	67	2	1	409	14	5	150	8	3	190	7	4
Age and sex-adjusted HR (95% CI)	1	————	————	1	1.23(0.72-2.10)	0.96(0.40-2.31)	1	2.03(0.98-4.17)	1.29(0.41-4.06)	1	0.88(0.41-1.89)	1.14(0.42-3.08)
Multivariable HR (95% CI)*	1	————	————	1	1.09(0.63-1.87)	0.86(0.35-2.08)	1	1.73(0.83-3.61)	1.24(0.39-3.95)	1	0.84(0.39-1.83)	1.28(0.47-3.51)
Multivariable HR (95% CI)†	1	————	————	1	1.04(0.60-1.79)	0.82(0.34-2.00)	1	1.63(0.77-3.45)	1.22(0.38-3.93)	1	0.86(0.39-1.86)	1.21(0.44-3.34)
**Total cardiovascular disease, n**	439	17	8	2007	57	25	749	35	10	780	26	12
Age and sex-adjusted HR (95% CI)	1	1.56(0.96-2.55)	1.63(0.81-3.28)	1	1.03(0.79-1.34)	0.99(0.66-1.47)	1	1.78(1.26-2.50)	0.89(0.48-1.67)	1	0.92(0.62-1.37)	0.94(0.53-1.66)
Multivariable HR (95% CI)*	1	1.54(0.94-2.54)	1.71(0.84-3.48)	1	0.97(0.75-1.27)	0.92(0.62-1.36)	1	1.72(1.21-2.43)	0.86(0.46-1.62)	1	0.92(0.61-1.36)	0.96(0.54-1.72)
Multivariable HR (95% CI)†	1	1.54(0.93-2.56)	1.59(0.77-3.26)	1	0.97(0.74-1.27)	0.89(0.06-1.32)	1	1.71(1.20-2.43)	0.89(0.47-1.67)	1	0.90(0.60-1.34)	0.93(0.52-1.66)

* Adjusted further for histories of hypertension and diabetes, smoking status, alcohol intake, hours of walking, hours of sports, hours of sleeping, educational status, perceived mental stress, and marital status.

† Adjusted further for food and nutrients: skipping breakfast, total energy intake, food intakes of fish, fruits, vegetables, and nutrients intakes of sodium and saturated fatty acids.
